# Piperacillin-Tazobactam-Induced Generalized Exanthematous Pustulosis

**DOI:** 10.7759/cureus.83786

**Published:** 2025-05-09

**Authors:** Sudeep Chapagain, Prabina Basnet, Surendra Khanal, Zahraa Rabeeah, Njika Atemnkeng

**Affiliations:** 1 Internal Medicine, Piedmont Athens Regional Medical Center, Athens, USA

**Keywords:** acute generalized exanthematous pustulosis, beta-lactam, beta-lactam antibiotics, cutaneous drug reaction, fever, piperacillin-tazobactam

## Abstract

Acute generalized exanthematous pustulosis (AGEP) is an extremely rare condition that is mostly caused by antibiotic exposure. Clinically, it manifests as the rapid appearance of multiple sterile pustules, swelling, and erythema within a few hours to days of exposure to the offending medication. Skin lesions usually resolve by desquamation after discontinuation of the offending agent. We present a case of a 58-year-old female patient with AGEP in the setting of piperacillin-tazobactam use, which was confirmed by histopathology with findings of neutrophilic pustules and eosinophilic infiltration. Her symptoms started resolving within three days of stopping the medication.

## Introduction

The nomenclature of acute generalized exanthematous pustulosis (AGEP) was first proposed by Baker and Ryan in 1968 [1]. Although the vast majority of reported cases are due to adverse drug reactions to commonly used medications like β-lactams and macrolides, it is a rare condition with an estimated incidence of one to five cases per million per year [2,3]. It is generally thought to be due to a T-cell-mediated hypersensitivity reaction with a sterile neutrophilic inflammatory response, mostly to drugs. However, the exact pathophysiology is unknown [2]. The American Academy of Allergy, Asthma, and Immunology describes it as the appearance of multiple sterile pustules occurring in association with swelling and redness as an acute skin reaction to certain medications that occurs within a certain number of days of medication exposure. With subsequent typical desquamation, it usually resolves within 15 days with an overall good prognosis. Lesions mostly occur in intertriginous areas, with mucosal involvement in around 20% of cases. Fever and leukocytosis often accompany the skin lesions [4].

## Case presentation

A 58-year-old female with a decubitus ulcer secondary to chronic multiple sclerosis presented with fever and a generalized itchy, painless rash for three days. She was being treated for coccygeal osteomyelitis with piperacillin-tazobactam for 22 days before this presentation and was still receiving the same medication. No similar rash was reported on close contacts.

**Figure 1:**
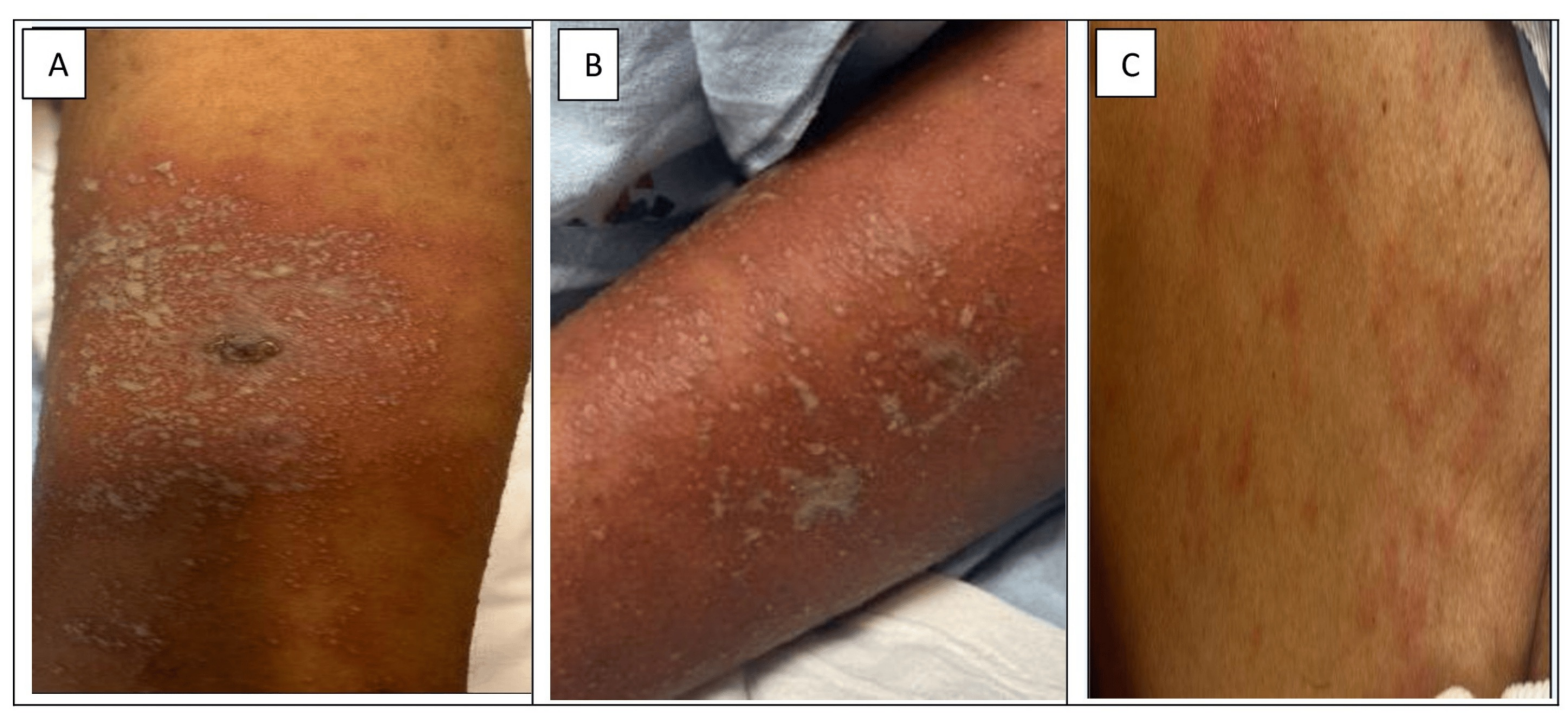
Maculopapular and pustular rash over bilateral forearms (A, B) and trunk (C) from left to right

**Figure 2:**
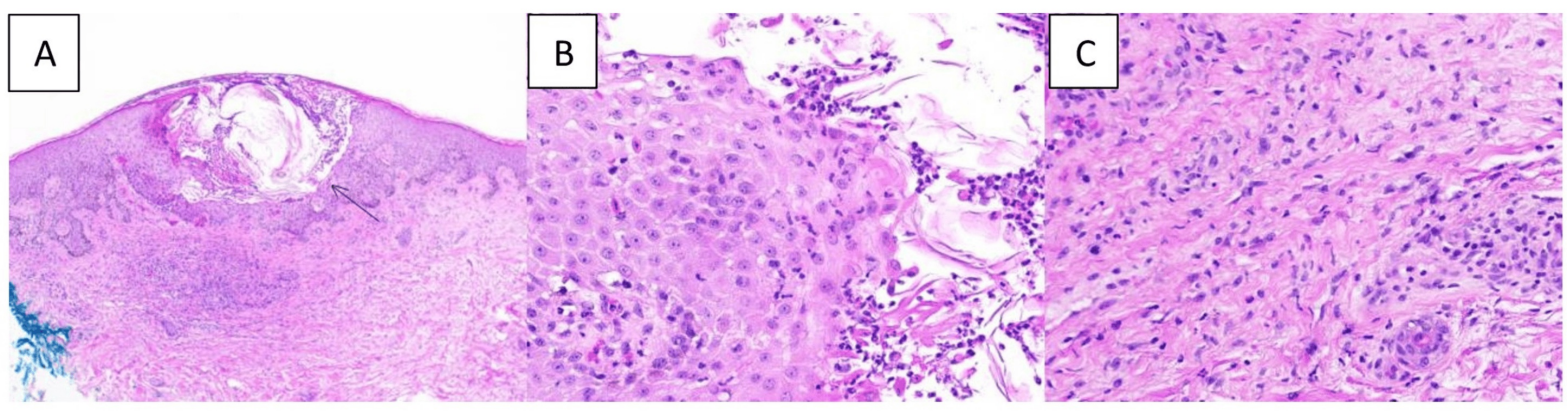
Biopsy images of the skin lesion from the right leg (A) The arrow shows a pustule in the epidermis; (B) neutrophils in the epidermis and a few necrotic keratinocytes; (C) eosinophils in the dermis

The physical examination showed a generalized maculopapular and pustular rash present over the neck, bilateral forearms, bilateral thighs, and legs (Figure 1). There was no involvement of the mucosal membranes. Blood work showed leukocytosis, elevated eosinophils (10% on manual diff), and elevated CRP. Urine culture and blood culture were negative. A punch biopsy was done from the right lower leg, and the histopathology sample showed neutrophils in the pustule and in the adjacent epidermis, neutrophils in the epidermis, and a few necrotic keratinocytes and eosinophils in the dermis, which was suggestive of AGEP (Figure 2). Direct immunofluorescence was negative for IgG, IgG4, IgA, IgM, C3, C5b-9, and fibrinogen deposition. There was no immunofluorescence evidence of connective tissue disease, vasculitis, dermatitis herpetiformis, porphyria cutanea tarda, pseudo-porphyria, or autoimmune blistering disease.

She was admitted and started on supportive care along with discontinuation of medicine. She received acetaminophen as needed for fever. The fever and leukocyte count gradually improved. We did not use steroids (either topical or systemic) for our patient.

The discontinuation of piperacillin-tazobactam caused pustular blisters to coalesce and revealed areas of desquamation over the papules. She was discharged after three days of hospital admission, and during the discharge, most of her lesions were coalesced with desquamation. The lesions resolved completely by 10 days. She was informed that she should avoid piperacillin-tazobactam transfusions in the future, and the condition was documented as an allergy in her documentation.

## Discussion

In this case, the patient had a history of taking piperacillin-tazobactam (a ureidopenicillin) for the treatment of chronic coccygeal osteomyelitis and presented with clinical evidence of generalized maculopapular and pustular lesions, accompanied by fever and leukocytosis. In this case, the latency period of approximately three weeks between the initiation of the offending agent and the appearance of the rash aligns with the typical onset of AGEP. Generalized pustular psoriasis and acute generalized pustulosis were considered as the differential diagnoses. A punch biopsy from the lower leg showed features of AGEP, which helped us confirm the diagnosis. A rapid resolution of the symptoms, including the cutaneous lesions, was observed after stopping the suspected offending agent.

AGEP is one of the types of adverse cutaneous drug reactions that can start approximately 4-14 days after starting the drugs. Clinical features mainly include a generalized erythematous eruption with superficial desquamation. It can be associated with fever and small sterile pustules, which are found mainly in the axillary and inguinal areas. It is most commonly associated with antibiotics. Agents include penicillin (aminopenicillins and ureidopenicillins), quinolones, terbinafine, diltiazem, hydroxychloroquine, isoniazid, and sulfonamides. Management mainly includes stopping the offending drugs. It has a good outcome and usually settles within two weeks of stopping the agent. Other supportive therapy includes the use of emollients and topical steroids if needed [5]. A retrospective study conducted in Thailand identified systemic drugs as the most common offending agents, with antibiotics constituting 75% of the cases. Among the antibiotics, β-lactam was the most common agent [6].

One of the main differential diagnoses is pustular psoriasis, which can be differentiated via histopathology. Dermal edema and spongiosis in the background, along with perivascular eosinophils and subcorneal or intraepidermal pustules, may be seen in AGEP, which were also the findings in our case. The absence of hyperplasia and acanthosis helps differentiate it from pustular psoriasis [7]. The histopathology in our case also showed subcorneal pustules with neutrophils and dermal eosinophils, which helped us to differentiate it from pustular psoriasis, where neutrophil predominance would be expected rather than neutrophils with eosinophils. Additionally, other differentials such as DRESS (drug reaction with eosinophilia and systemic symptoms), SJS (Stevens-Johnson syndrome), and TEN (toxic epidermal necrolysis) were initially considered, as these could also be associated with antibiotics. However, the absence of mucosal involvement made SJS/TEN less likely. Our patient had a pustular lesion, which is rarely seen in DRESS.

AGEP is a benign condition and is self-limiting [8,9]. There is no specific treatment for AGEP. The treatment for AGEP primarily involves discontinuing the causative agent and providing supportive care to prevent secondary infections. Antipyretics can be used for fever, and antihistamines can be used [10,11]. For itching, topical steroids have also been used for severe pruritus. Systemic steroids are not used routinely, but short courses of up to five days can be used in severe cases [12].

## Conclusions

In day-to-day practice, we use various antibiotics to treat infections, with aminopenicillins being one of the most commonly used groups of antibiotics, which are among the main culprits for AGEP. Although it is a rare adverse reaction, whenever we encounter a patient on ureidopenicillin/aminopenicillin with fever and pustular rash, we should consider AGEP, as the immediate discontinuation of the offending agent is the mainstay of management. It usually has a benign course and resolves on its own. Histopathology helps confirm the diagnosis and rule out other conditions, such as pustular psoriasis, that can mimic AGEP.
